# Development of the *piggyBac *transposable system for *Plasmodium berghei *and its application for random mutagenesis in malaria parasites

**DOI:** 10.1186/1471-2164-12-155

**Published:** 2011-03-20

**Authors:** Jannik Fonager, Blandine MD Franke-Fayard, John H Adams, Jai Ramesar, Onny Klop, Shahid M Khan, Chris J Janse, Andrew P Waters

**Affiliations:** 1 Leiden Malaria Research Group, Department of Parasitology, Leiden University Medical Center, Albinusdreef 2, 2333 ZA Leiden. The Netherlands; 2 Department of Global Health, College of Public Health, University of South Florida, Tampa, Florida USA; 3 Institute of, Infection, Immunity & Inflammation, School of Medical, Veterinary & Life Sciences, & Wellcome Centre for Molecular Parasitology, Glasgow Biomedical Research Centre, University of Glasgow, Scotland, UK

## Abstract

**Background:**

The genome of a number of species of malaria parasites (*Plasmodium *spp.) has been sequenced in the hope of identifying new drug and vaccine targets. However, almost one-half of predicted *Plasmodium *genes are annotated as hypothetical and are difficult to analyse in bulk due to the inefficiency of current reverse genetic methodologies for *Plasmodium*. Recently, it has been shown that the transposase *piggyBac *integrates at random into the genome of the human malaria parasite *P. falciparum *offering the possibility to develop forward genetic screens to analyse *Plasmodium *gene function. This study reports the development and application of the *piggyBac *transposition system for the rodent malaria parasite *P. berghei *and the evaluation of its potential as a tool in forward genetic studies. *P. berghei *is the most frequently used malaria parasite model in gene function analysis since phenotype screens throughout the complete *Plasmodium *life cycle are possible both in vitro and in vivo.

**Results:**

We demonstrate that *piggyBac *based gene inactivation and promoter-trapping is both easier and more efficient in *P. berghei *than in the human malaria parasite, *P. falciparum*. Random *piggyBac*-mediated insertion into genes was achieved after parasites were transfected with the *piggyBac *donor plasmid either when transposase was expressed either from a helper plasmid or a stably integrated gene in the genome. Characterization of more than 120 insertion sites demonstrated that more than 70 most likely affect gene expression classifying their protein products as non-essential for asexual blood stage development. The non-essential nature of two of these genes was confirmed by targeted gene deletion one of which encodes P41, an ortholog of a human malaria vaccine candidate. Importantly for future development of whole genome phenotypic screens the remobilization of the *piggyBac *element in parasites that stably express transposase was demonstrated.

**Conclusion:**

These data demonstrate that *piggyBac *behaved as an efficient and random transposon in *P. berghei*. Remobilization *of piggyBac *element shows that with further development the *piggyBac *system can be an effective tool to generate random genome-wide mutation parasite libraries, for use in large-scale phenotype screens *in vitro *and *in vivo*.

## Background

The sequencing of several *Plasmodium *genomes [1-4] has permitted large-scale microarray and proteomic studies of the different *Plasmodium *life cycle stages [3,5-8]and comparative genomic analyses [9-12]. These studies have generated a wealth of information on the majority of the ~5.500 *Plasmodium *genes and have provided insight into the timing of expression during the lifecycle and into the putative function of many of the encoded proteins. However, almost one-half of the predicted genes still lack characterized orthologues in other systems and for most of these genes, the function remains unknown and the gene model unconfirmed. Reverse genetic approaches are often used to assign function to *Plasmodium*-specific genes. However, larger scale gene function analysis using reverse genetics in *Plasmodium *is hampered by the relative inefficiencies of genetic modification by targeted gene disruption or mutation [13,14] and by the absence of other methods to modify gene expression such as RNAi gene silencing [15]. Forward genetic approaches have not been widely applied in *Plasmodium *research because of the lack of adequate tools for whole genome analysis. Forward genetics is an experimental approach in which gene mapping and positional cloning are used to elucidate the molecular mechanisms underlying phenotypic differences between two individuals for a given trait. The advantage of this approach is that it involves an unbiased/random sampling of the genome, screening for a pre-determined phenotypic trait offering the possibility to identify multiple genes associated with a trait that directed reverse genetics is less likely to do. Often phenotypes are intentionally created by random mutagenesis using chemicals, radiation or insertional mutagenesis. Insertional mutagenesis has been at the core of functional genomics in many species. Transposable elements have been widely used to induce insertional mutagenesis in highly diverse biological systems and remain a mainstay for important model organisms. In a direct comparison of the four different transposable systems *Sleeping-beauty*, *Tol2*, Mos1 and *piggyBac *in four mammalian cell lines, *piggyBac *demonstrated significantly higher transposition activity in all lines [16]. In *Plasmodium*, transposition has been reported using the *Drosophila mariner *transposable element but the transposition events occurred at a very low frequency independent of transposase [17]. Recently, the *piggyBac *system has been successfully adapted for the human malaria parasite *P. falciparum *through the use of a two plasmid transfection approach: one transiently maintained plasmid containing the transposase and the other plasmid containing a positive selectable marker expression cassette flanked by the Inverted Terminal Repeat (ITR) sequences necessary for transposase mediated insertion. Parasites containing successful insertion events are drug-selected and this approach has enabled parasite efficient transformation by the *piggyBac *element [13,18]. *PiggyBac *has now been successfully applied as a forward genetics tool using phenotypic screening of pools of *P. falciparum *mutants to identify genes that play a role in asexual blood stage development [19].

In this study we report the development of the *piggyBac *transposition system for the rodent malaria parasite *P. berghei*. The availability of relatively efficient reverse genetic technologies for *P. berghei *and the fact that these can be combined with analyses on parasites throughout their complete life cycle, both *in vitro *and *in vivo*, have made *P. berghei *the most frequently used model for gene function analysis [20-22]. The development of additional tools for analysis of gene function that would allow larger scale experiments would enhance gene function analysis in *Plasmodium*. The use of insertional mutagenesis as a tool for larger scale analysis of gene function is dependent on the efficiency of random insertion which in turn is dependent on: 1) the transfection efficiency of parasites for introducing the two plasmids, the *piggyBac *donor plasmid and the transient helper plasmid containing the transposase, in co-transfection experiments, 2) the activity in *Plasmodium *of a transposase which is evolved to function in insect cells and 3) the genome wide frequency, distribution and accessibility of the target TTAA site of integration. The *P. berghei *genome, like that of *P. falciparum*, is one of the most AT-rich of all eukaryotic genomes characterized (> 80% AT rising to >90% in non-coding and centromeric regions [1,10]. Indeed, the *P. falciparum *genome is estimated to possess more than 300,000 TTAA sites with ~40% appearing in all characterized ESTs yielding an average of >20 integration sites per gene [13]. Moreover, for *P. berghei *efficient transfection methods are available [23]. Co-transfection of the transient helper plasmid and donor plasmid (as was also performed in *P. falciparum*), is commonly used to prevent unwanted *piggyBac *remobilization in the genome. However, it has been shown that constitutively active or regulated transposase expression can improve transposition efficiency up to 6 times [24]. In this study we have investigated the frequency of random integration in parasites by expressing the transposase either transiently from introduced episomes or 'constitutively' from a transposase gene integrated into the genome. We observed efficient and random *piggyBac*-mediated insertion into the genome when parasites were transfected with *piggyBac *donor plasmids under both circumstances; either in combination with transposase-containing helper plasmids or in parasites containing the transposase gene stably integrated into the genome. We present evidence for *piggyBac *element remobilization in the latter parasites and therefore such parasites can be effective tools for generation of mutant parasite libraries containing random mutations. The availability of the technology for transposon-mediated random mutagenesis for *P. berghei *can be used to develop and apply large-scale forward genetic screens for analysing gene function.

## Results

### Generation of *piggyBac *donor and helper plasmids and a transgenic *P. berghei *line containing transposase in its genome

**Figure 1:**
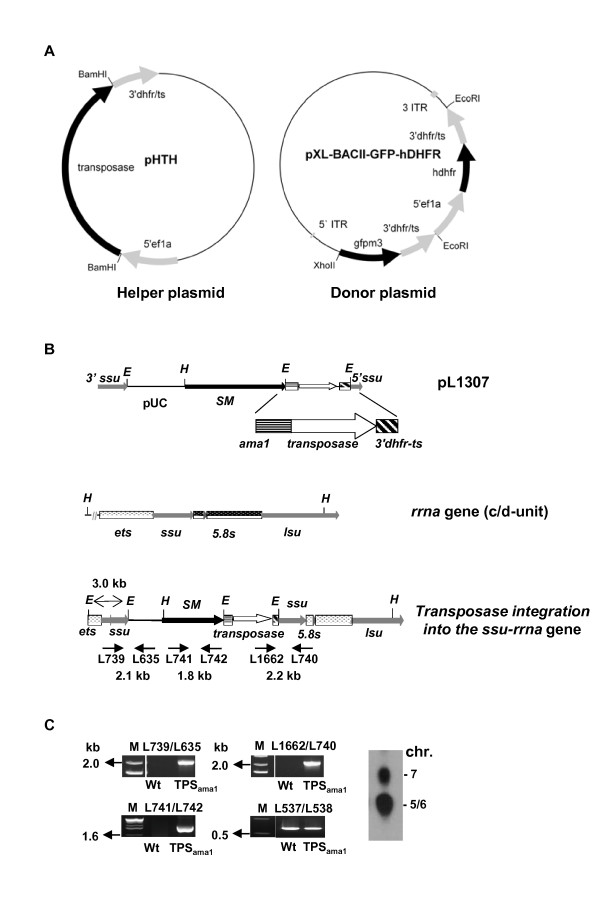
**The *piggyBac *insertion (donor) construct and helper constructs for expression of transposase**. **A**. The helper plasmid pHTH (pL1301, left) contains the *transposase *gene under control of the constitutive *eef1aa *promoter and the *dhfr/ts 3'UTR *for transient transposase expression and the donor plasmid (pL1302, right) contains the *gfp*-expression cassette without a promoter and the *hdhfr *selectable marker cassette. Both cassettes are flanked by the *piggyBac *inverted terminal repeats (ITR's). **B**. Schematic representation of the construct pL1307 for stable integration of the transposase gene (under the control of the *ama1 *promoter) into the *P. berghei *genome in the non-essential small subunit ribosomal rna gene (*ssu-rrna*) of the *c/d-rrna *unit. SM: the *tgdhfr/ts *selectable marker cassette. Primers used for diagnostic PCRs are indicated by arrows with the expected fragment size (see **C**). *lsu*: large subunit, *ets*: external transcribed spacer region. **C**. Diagnostic PCR and FIGE analysis of separated chromosomes of mutant TPS_ama1 _confirming correct integration of construct pL1307 into the *rrna *gene locus. See **B **for the location of the primers; 537/538 control primers for the *p28 *locus; (Additional file 3 Figure S1).

Two different approaches were used to achieve insertion of *piggyBac *elements into the genome of *P. berghei*. In the first approach parasites were simultaneously 'co-transfected' with *piggyBac *donor plasmid (pL1302) and helper plasmid (pL1301). The helper plasmid contains the transposase under the control of the constitutive *eef1a *promoter but does not contain a drug-selection cassette (Figure 1A). Since such plasmids are not retained in parasites during asexual growth without drug-selection [25] the helper plasmid is 'transiently transfected' and will be lost from the parasites during blood stage growth. In the second approach the donor plasmid was transfected into transgenic parasites that contained 2 copies of the transposase stably integrated into the *c-ssu-rrna *gene locus (Figure 1C). This transgenic line, *transposase ama-1 *(abbreviated: TPS_ama1_), was generated using standard methods for transfection of *P. berghei *and the transgenic parasites contain the *T. gondii dhfr/ts *as a selectable marker and *transposase *under the control of the schizont specific *ama-1 *promoter (Figure 1B). The construct was integrated into the *c-ssu-rrna *gene locus by single cross-over integration, resulting in the integration of 2 copies of the transposase gene.

The donor plasmid contains the 5'and 3' inverted terminal repeats of the *piggyBac *element (Figure 1A) which are the minimal *cis *elements necessary for *piggyBac *mobilization. Both inverted repeat sequences consist of a terminal 13 bp and internal 19 bp perfect inverted repeat that are separated by a 3 bp (5'ITR) or a 31 bp (3'ITR) spacer [26-30]. In the donor plasmid the two ITR sequences are located on both sides of a drug-selectable marker cassette and a *gfp *expression cassette that lacks a promoter region (Figure 1A). The target site for *piggyBac *insertion is TTAA and it moves by precise insertion and excision mechanisms [31,32]. Transfection of the donor plasmid would therefore result in insertion of both the drug-selectable marker cassette and the *gfp*-expression. Insertion of the drug selectable marker, the human *dhfr *gene, allows for selection of parasites containing the inserts using pyrimethamine or WR99210.

### Transfection of donor plasmids into parasites that either transiently or stably express transposase results in *piggyBac*-mediated insertion

**Figure 2:**
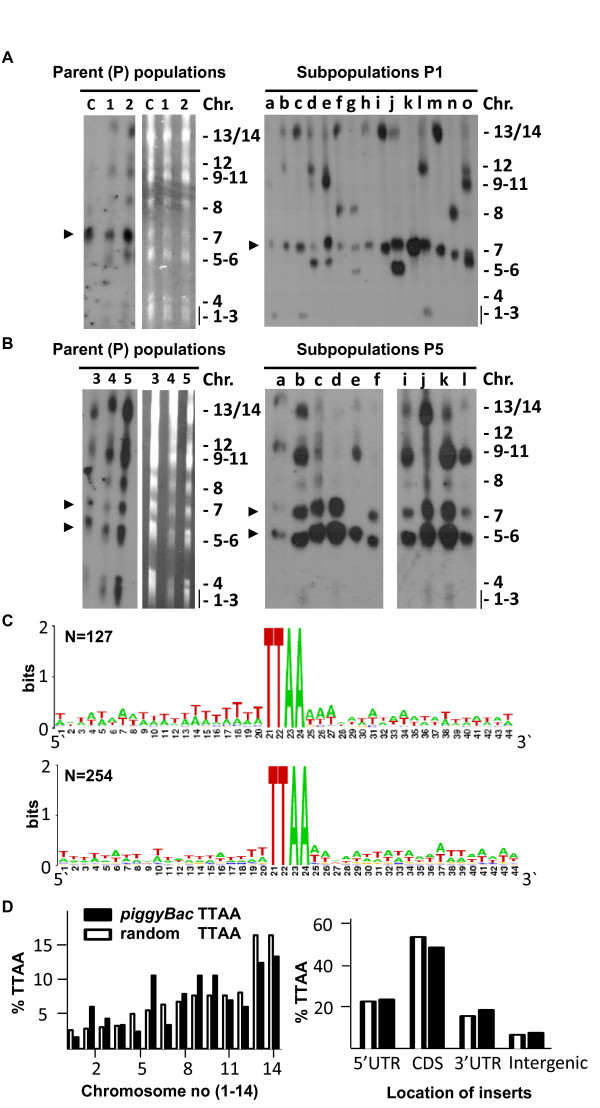
**Location of *PiggyBac *inserts into the genome of *P. berghei***. **A**. *PiggyBac *insertions as shown by FIGE analysis of separated chromosomes hybridized with the *pbdhfr/ts *probe. This probe recognizes the inserts and the endogenous *dhfr/ts *gene on chromosome 7 (arrow). Left panel: Inserts in two parent parasite populations (1, 2) after transient transfection of the helper (h) and donor (d) plasmid (h/d ratio: P1 = 1:1; P2 = 1:2; c = control parasites transfected with only donor plasmid). Right panel: insertions in 15 (a to o) parasite subpopulations of P1. **B**. *PiggyBac *insertions as shown by FIGE analysis of separated chromosomes hybridized with the *pbdhfr/ts *probe, which recognizes the endogenous *dhfr/ts *gene (chromosome 7) and the transposase construct pL1307 in chromosome 5 (arrows). Left panel: Insertions in the three parent parasite populations after transient transfection of the donor (d) plasmid into parasites of mutant TPS_ama1 _that contains transposase integrated into chromosome 5 (amount of d: P3 = 15 μg; P4 = 10 μg; P5 = 5 μg). Right panel: insertions in 10 parasite subpopulations of P5 (a to l). **C**. Upper panel: WebLogo representation of the sequence of 127 *piggyBac *insertion sites, showing the TTAA insertion site and 20 bp up- and downstream of the *piggyBac *5'ITR and 3'ITR, respectively. Lower panel: WebLogo representation of 20 bp up- and down-stream sequence of 254 randomly chosen TTAA sites in the *P. berghei *genome. **D**. Left panel: Chromosomal distribution of the 254 randomly chosen TTAA sites and 124 *piggyBac *TTAA insertion sites. Right panel: location of the *piggyBac *inserts (black bars) and 254 random TTAA sites (white bars) in CDS (+introns), within 1 kb 5' or 1 kb 3' to the CDS (designated as 5' UTR and 3 'UTR) or in the intergenic regions (> 1 kb from CDS).

The two approaches described above were used to obtain *piggyBac*-mediated insertion of the *gfp*-expression cassette into the *P. berghei *genome. Co-transfection of *wt *parasites with both the donor and the helper plasmid, followed by selection with pyrimethamine resulted in selection of two resistant parasite populations (1055 and 1056; parent populations P1 and P2, respectively). Southern analysis of Field Inversion Gel Electrophoresis (FIGE) -separated chromosomes of these parasites showed integration of the donor plasmid (construct) in multiple chromosomes (Figure 2A). No integration was detected when only donor plasmid was transfected (exp. 1057, Figure 2A, lane C). Based on hybridization intensity it appears that a ratio of helper/donor plasmid of 1:2 results in higher insertion frequency then a 1:1 ratio (Figure 2A). To obtain a better insight into the insertion into the different chromosomes we generated 15 'subpopulations' (P1a-P1o) by intravenous injection of 1-5 parasites of parent population P1 in 15 different mice. Southern analysis of FIGE-separated chromosomes of parasites from the subpopulations showed insertion of the constructs in nearly all chromosomes (Figure 2A, right panel).

Similarly, transfection of 3 different amounts (P3 = 15 μg; P4 = 10 μg; P5 = 5 μg) of the donor plasmid into TPS_ama1 _parasites that stably express transposase, followed by selection with WR992210, resulted in selection of three resistant parasite populations (1182cl1m1-m3; parent population P3, P4 and P5). Southern analysis of FIGE-separated chromosomes of these parasites again showed integration of the donor plasmid (construct) in multiple chromosomes (Figure 2A, left panel). Based on the relative intensity of the hybridization signals it appears that a lower concentration of the donor plasmid results in higher insertion frequency. Analysing the Southern hybridization data of the FIGE-separated chromosomes from the 10 subpopulations of P5 (generated as described above) insertion of the constructs was observed in nearly all (groups) of chromosomes (Figure 2B, right panel).

### *PiggyBac *insertion into all chromosomes

To identify the location of *piggyBac *insertions in the genome we initially used a standard method of inverse PCR using the forward primers 3202-3204 in combination with the reverse primers 3205-3207 as described for *P. falciparum *[18] to amplify re-ligated *piggyBac *sequences with *P. berghei *genomic flanking regions. However, this PCR method resulted in a very low yield of inserts when we used DNA extracted from parasites of the subpopulations. Compared to the number of insertions estimated based on the number positive chromosomes, we were able to retrieve less than 20% of the inserts from the different subpopulations (data not shown). We therefore decided to use an adapted method of TAIL-PCR. In this method a large pool of arbitrary degenerate primers designed for use in the AT rich genome of *P. berghei *(see Methods section) were used in combination with primers specific for both ITR's of the *piggyBac *element. By analyzing 9 subpopulations that had 16 visible inserts in the 14 *P. berghei *chromosomes, we were able to identify 11 inserts (~70%) from these chromosomes by TAIL-PCR. Therefore, for further identification of inserts we decided to exclusively use TAIL-PCR and no other methods such as inverse PCR or methods using restriction digestion and ligation [33]. Using TAIL-PCR we identified insertions in parasites of parent population 2 and 5 and subpopulations of P1, P2 and P5. In total we identified 127 inserts at unique locations in the genome (Additional file 1 Table S1) and we have not identified any insert at the same location from two different parent populations, suggesting that there is no strong bias in preferred insertion sites between different experiments. However, for two genes: [GeneDB[34]:PBANKA_060790] (in P2 and P5.b.3) and [GeneDB[34]:PBANKA_082890] (in P2 and P5.i.3), insertions were identified both in 5'UTR/3'UTR and in CDS/3'UTR, respectively. In all 127 cases, that we identified by sequencing TAIL-PCR products, the insertion occurred via an expected canonical TTAA tetranucleotide (*piggyBac*) insertion site (Figure 2C, Additional file 1 Table S1).

Since the ability of *piggyBac *to randomly insert into the genome is an important feature of the *piggyBac *mutagenesis system in *Plasmodium *research, we investigated several aspects of the 127 insertions. First, we analysed the immediate 20 nucleotides adjacent to 5´ and 3´ flanking the TTAA site to evaluate if *piggyBac *insertion exhibited any additional preferences within the insertion flanking sequences. We compared these flanking regions with those of a set of 254 random chosen TTAA sequences from the genome (see Methods section). Overall, a slightly higher AT% was found in the *piggyBac *TTAA site flanking regions (81.7%) compared with the 254 random TTAA sites (80.4%), however this difference was not statistically significant (two tailed test, P = 0.13). A slight preference for a stretch of seven T's and three A's was observed at the 5´ and 3' of the TTAA *piggyBac *insertion sites (Figure 2C) when compared to the flanking regions of the randomly selected TTAA sites (Figure 2C). Both the 5'-stretch of seven T's and the 3'stretch A's at the *piggyBac *TTAA insertion site were significantly different from similar stretches of the random chosen TTAA sites (Chi Square: 5,5E-08 *** and 0,013* for the 5'and 3' stretches, respectively). This indicates that *piggyBac *may have a slight insertional bias with regard to the TTAA flanking sequences. We next analysed the chromosomal distribution of the 127 inserts. Three inserts (we term repetitive region 1-3) could not be mapped to a specific chromosome but were located on contigs containing genes that belong to known subtelomeric gene families, the *bir *or *Pb-fam *families of genes [10,35]. The 124 remaining inserts were spread over all 14 chromosomes (Figure 2D, left panel) and a weak correlation (R^2^: 0.56) was observed between the number of inserts and the size of the chromosome. Chromosome 6 was exceptional as more than expected inserts (13) were identified in this chromosome compared to the number identified in the similar sized chromosomes 5, 7 and 8 in which 4-10 inserts were identified.

### *PiggyBac *insertions into coding, untranslated and intergenic regions

The position of the 127 inserts in and around predicted genes show that *piggyBac *inserted both in coding sequence (CDS) and untranslated regions (UTR's) of genes (Figure 2D, right panel). For these regions a comparable proportion of *piggyBac *TTAA insertion sites and 254 randomly chosen TTAA sequences were observed: 24% (piggyBac integration) and 23% (random TTAA sites) in 5'UTR's; 45% and 54% in CDS (including introns); 23% and 16% in 3'UTRs; 8% and 7% in intergenic regions. For all regions the distribution of the *piggyBac *insertion sites was not significantly different from the random selected TTAA sites (Fisher's test: 5'UTR: p = 0.90, CDS (including introns): p = 0.15, 3'UTR: p = 0.12; intergenic regions: p = 0.84). These results indicate that *piggyBac *insertion in *P. berghei *occurs randomly and there is no preference for insertion either within the CDS or non-CDS of genes. We analysed whether *piggyBac *had a preference for insertion into transcribed/expressed genes, by analyzing published data on expression of genes with *piggyBac *insertions. For this analysis we used the new *P. berghei *gene models/systematic id's provided by the 'GeneDB 2010 release' and included only insertions that were located at a distance of more than 1 kb from the CDS (see Additional file 1 Table S1 for the included/excluded genes). Expression data were obtained from published proteomes from different *P. berghei *life cycle stages [3] and gametocytes [7]. In addition, all available *P. berghei *EST databases present in the PlasmoDB database [36] were used (using the gene overlap function at default settings to retrieve ESTs matching genes assigned to different life cycle stages). Of the 124 analysed *piggyBac *inserts, 91 (73%) had proteome/EST based expression evidence compared with expression assigned to 3594 of the 4479 (80%) *P. berghei *gene models ('2010 Sanger *P. berghei *release'). Of the genes with *piggyBac *inserts, 55% (68 of 124) were transcribed/expressed in the asexual blood stages (ABS) compared to 54% of all *P. berghei gene *models (2420 of 4479) with evidence for transcription/expression in ABS (not significant, Fishers test: p = 0.07). Since genes that are inactive in ABS might be less accessible for *piggyBac *insertion, we compared the proportion of inserts in genes which are inactive in ABS but are expressed in other stages. There is transcription/proteome evidence that 26% (1174 of 4479) of all genes are expressed exclusively in stages other than the ABS. For genes with *piggyBac *inserts, 20% (25 of 124) of the genes are expressed in other life cycle stages but not in ABS (not significant, Fishers test: p = 0.15). In none of the comparisons mentioned above were significant differences found, indicating that *piggyBac *insertion is not linked to the expression pattern or activity of the genes.

A large proportion of the inserts were found inside CDS, CDS introns or in the 5'UTR regions (86 out of 127) of genes and such insertions most likely affect or completely disrupt the expression of these genes. The presence of an insert in the CDS/introns/5'UTR may therefore provide indirect evidence that the gene is not essential for ABS development. This is because *Plasmodium *is haploid during ABS development; therefore disruption of a gene essential for ABS would result in parasites that can not be selected after transfection as the deletion is lethal. We analysed 73 genes in more detail that contained inserts either in the CDS, CDS intron or in the 5'UTR with a maximum distance of 500 bp to the start codon of the CDS (Additional file 2 Table S2). Interestingly for at least 4 genes evidence already existed that expression of these genes is not essential in ABS as have been demonstrated by standard targeted deletion of these genes (See references in Additional file 2 Table S2). In addition, published data on expression in blood and mosquito stages [7,8] showed that 8 genes (or their *P. falciparum *orthologs) are either gametocyte- or mosquito-stage specific and are not expressed in ABS, indicating that the lack of expression should have no effect on the survival of ABS. However, for many genes with *piggyBac *inserts evidence is available indicating expression of these genes in ABS (collated and available at PlasmoDB [36]) suggesting that these genes have a non-essential and/or redundant function during asexual blood stage development. We confirmed the non-essential role of two of such genes, *p41 *([GeneDB [34]:PBANKA_100260]; interrupted in its 5' UTR by *piggyBac*) and *metacaspase2 *([GeneDB [34]:PBANKA_130230]; interrupted in a CDS intron by *piggyBac*), through targeted disruption of these genes. P41 belongs to proteins encoded by the 6-cysteine family of genes and in *P. berghei *is transcribed in ABS [37]. In *P. falciparum *P41 is intimately associated with GPI-anchored proteins that are located to the surface of merozoites (i.e. in lipid rafts). It has been reported that all *P. falciparum *GPI-anchored proteins (apart from MSP5) on the surface of merozoites are essential [38-40]. We targeted *P. berghei p41 *by disruption of the CDS through double cross-over recombination (Additional file 3 Figure S1) and confirmed that this protein was not essential in *P. berghei *ABS. The gene encoding *P. falciparum metacaspase 2 *[PF14_0363] is transcribed throughout ABS development [36,41]. As with P41, we were able to disrupt the CDS of *P. berghei metacaspase2 *by targeted double cross-over recombination (Additional file 3 Figure S1), confirming the non-essential role of this protein in ABS. These data indicate that analysis of large scale *piggyBac *mutagenesis can provide evidence for the dispensability of certain, albeit ABS-expressed, genes for *Plasmodium *ABS. All 73 genes with *piggyBac *inserts in the CDS, CDS introns or in the 5'UTR region (within 500 bp from the start codon) have been deposited in the publically accessible database of genetically modified rodent malaria parasites (RMgmDB; [42,22]) and this information on *piggyBac *insertion is linked to the information on individual genes in PlasmoDB [36] and GeneDB [34]. For identification of the exact location of the insertion, the sequence of 20 bp up-and downstream of the TTAA sequence, is provided for all genes in RMgmDB [42] (See also Additional file 2 Table S2).

### Evidence for re-mobilization of *piggyBac *inserts in parasites stably expressing transposase

**Figure 3:**
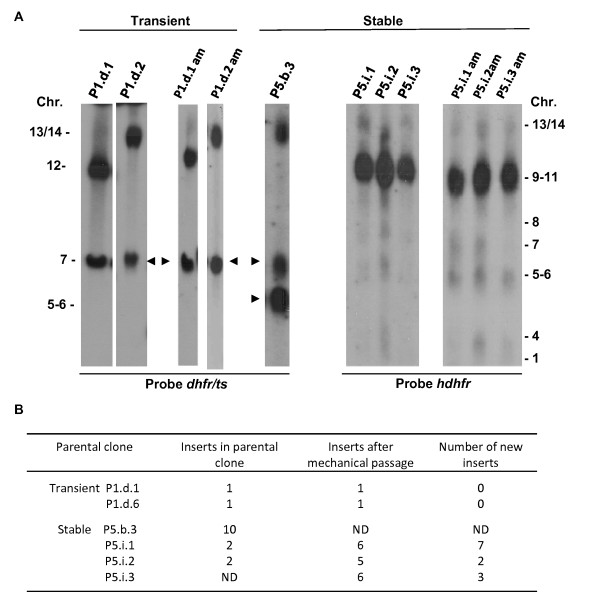
***PiggyBac *insertions in cloned parasites with transient or stable expression of transposase**. **A**. *PiggyBac *insertions as shown by FIGE analysis of separated chromosomes hybridized with the *pbdhfr/ts *or *hdhfr *probe. The *pbdhfr/ts *probe recognizes the inserted constructs, the endogenous *dhfr/ts *gene on chromosome 7 and the transposase construct pL1307 integrated into chromosome 5 (arrows). Without stable expression of transposase a single insert is detected in parasite clones both before and after prolonged periods of multiplication (i.e. after mechanical (am) passage; see **B**. In parasite clones that stably express transposase multiple inserts are detected in different chromosomes, both before and after mechanical passage (see B). **B**. Number of *piggyBac *inserts as determined by TAIL PCR in cloned parasite lines before and after prolonged periods of multiplication (i.e. after mechanical passage). ND: No data.

To test the stability of *piggyBac *inserts in the genome, we generated parasite clones from both the subpopulations P1 (transient transfection of transposase) and P5 (stable expression of transposase) by the method of limiting dilution. For cloning of both P1 and P5 parasites we performed two sequential cloning procedures in order to obtain 'pure clones'. For P1 this resulted in generation of two clones P1.d.1 and P1.d.2. For P5 we obtained 4 clones P5.b.3, P5.i.1, P5.i.2 and P5.i.3. For both clones of P1 FIGE analysis of separated chromosomes showed evidence for *piggyBac *insertion into a single chromosome (chromosome 12 and 13/14 respectively; Figure 3A). Also by TAIL-PCR analysis only one insertion was detected for both clones. One being 633 bp into the 3'UTR of the gene [GeneDB [34]:PBANKA_132830], located on chromosome 13 (clone P1.d.2) and the other being a mapped to a repetitive region, Repetitive region 1 (clone P1.d.1; see Additional file 1 Table S1). After mechanical passage of parasites of both clones for a period of 2 weeks (14 asexual multiplication cycles), no new insertions were detected by FIGE analysis of chromosomes (Figure 3A) and TAIL-PCR also identified only the same single insertion in each parasite clone as identified before mechanical passage (Additional file 1 Table S1). In contrast, in clones obtained from P5 lines that stably express transposase we found evidence for remobilization of the *piggyBac *insert, both by FIGE analysis of separated chromosomes and by TAIL-PCR. In clone P5.b.3 a dominant insert is observed in chromosome 13/14 (Figure 3A) but by TAIL-PCR we were able to identify 10 different inserts in different chromosomes (Figure 3B and Additional file 1 Table S1). Also in the three clones P5.i.1-3 one dominant insert was observed in the group of chromosomes 9-11 but faint hybridization signals are also observed in other chromosomes (Figure 3A), indicating the presence of additional inserts. TAIL-PCR confirmed the presence of inserts into chromosomes 9-11 as well as additional inserts in a number of different chromosomes in these three cloned lines (Additional file 1 Table S1). These results indicate that parasites of clones derived from the transposase parasite line are not homogeneous populations with respect to *piggyBac *inserts but they consist of mixed populations of parasites containing inserts in different chromosomes. After mechanical passage of parasites of the three P5.i.1-3 clones for a period of 2 weeks (14 asexual multiplication cycles), we detected by both TAIL-PCR and FIGE analysis 12 novel inserts within the parasite populations (Figure 3B, Additional file 1 Table S1). Together these results indicate that in the presence of transposase the *piggyBac *inserts are able to remobilize in the *Plasmodium *genome, as has been also reported in *Drosophila melanogaster, Bombyx mori *and mouse embryonic stem cells. [43-45]. In the absence of transposase remobilization was not detected and inserts remained stably integrated in the genome.

### The use of *piggyBac *to 'trap' *Plasmodium *promoters active in asexual blood stages

**Figure 4:**
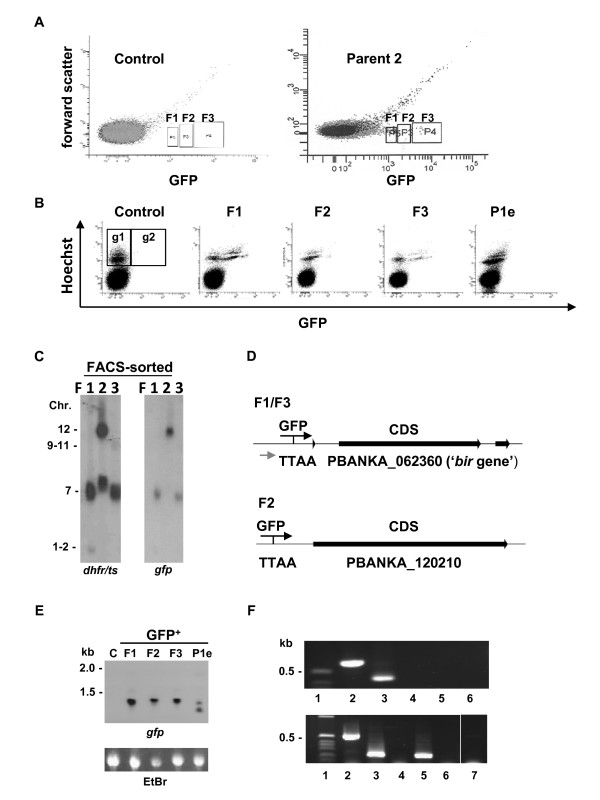
**Identification of *piggyBac*-trapped promoters**. **A**. FACS sorting of GFP-expressing parasites from a population with *piggyBac *inserts. Dot plots show GFP fluorescence and forward light scatter of (infected) erythrocytes of a control population without inserts (left plot) and of parent population P2 (see Figure 2A) with *piggyBac *inserts (right plot). Three populations (F1-F3) of GFP-expressing parasites were collected by sorting from gates F1 - F3. **B**. GFP-expression in blood stages of FACS-sorted populations F1-F3 and P1.5e (see Figure 2A). Gates: g1: infected erythrocytes (Hoechst positive) that are GFP-negative; g2: infected erythrocytes (Hoechst positive) that are GFP-positive. Percentages (mean + st. dev.) of GFP-positive infected cells: Control) 0.1%; F1) 41.6% + 3; F2) 4.0% + 0.7; F3) 28.1% + 2.9; P1e) 1. 6% + 0.2). **C**. *PiggyBac *insertions in GFP-expressing parasites of the F1-F3 populations shown by Southern analysis of chromosomes hybridized with *pbdhfr/ts *and *gfp *probes. **D**. Schematic representations of two *piggyBac *insertion sites identified by TAIL-PCR in the GFP-expressing parasites of the F1-F3 populations. Insertion location is compatible with GFP expression (black arrows; see also Additional file 1: Table S1). Grey arrow: location of integration primer (4571). **E**. Northern analysis of *gfp*-expression in blood stages of the Control and F1-F3 populations and subpopulation P1.5e. Loading control: ethidium bromide stained RNA. **F**. Confirmation of *gfp *transcription from the *bir *gene promoter in population F1 by RT-PCR (upper panel; see also Methods section; gDNA and cDNA (+RT or -RT enzyme) were obtained from F1 or Control parasites). Lanes: 1) Marker; 2) gDNA-F1; 3) cDNA-F1; 4) gDNA-Control; 5) cDNA-Control; 6) No DNA. Control of cDNA quality (lower panel) was performed by PCR across the two introns of the gene PBANKA_133840 (GeneDB [34]]). Lanes: 1) Marker 2) gDNA-Control; 3) cDNA-Control; 4) No cDNA-Control; 5) cDNA -F1; 6) No cDNA-F1; 7) No DNA.

Experimental validation of *P. berghei *gene models would significantly improve annotation of the genome. Therefore, we designed the *piggyBac *donor plasmid for promoter trapping experiments by introducing the *gfp*-expression cassette without a promoter region next to the 5'ITR1 sequence. Integration of the construct downstream of a promoter that is active in blood stages would therefore result in GFP expression in blood stage parasites. We analysed GFP-expression by fluorescence microscopy of parasites of primary transfection populations P1 and P2 that were obtained by transfection with the donor and helper plasmid. In both populations low numbers of GFP-positive blood stage parasites (< 0.1%) were detected whereas in the control parasites that were transfected with only the donor plasmid no GFP-positive cells were found (Figure 2). To isolate GFP-expressing parasites from population P2, we performed FACS sorting of GFP-positive blood stages from tail blood of mice with asynchronous infections. Three populations (F1-3) each of 10-50 GFP-positive cells were FACS-sorted by setting three different gates based on GFP-fluorescence intensity as shown in Figure 4A. These parasites were intravenously injected into mice to generate expanded parasite populations for further analysis. FACS analysis of blood stages of the F1-F3 populations (obtained from overnight cultures to enrich for maturing schizonts) confirmed GFP expression in blood stages (Figure 4B) and demonstrates different GFP-expression patterns between the sorted populations. However, since the initial sorting of GFP-expressing parasites was performed using asynchronous blood stages from tail blood and the confirmation of GFP-expression was derived from cultured schizonts, the GFP-fluorescence intensities cannot directly be compared between the populations shown in Figure 4A and B. Southern analysis of separated chromosomes showed integration of the *gfp*-expression cassette into multiple chromosomes (7 and 1/2 in F1; 12 in F2; 7 and 9/11 in F3; Figure 4C). Using TAIL PCR we confirmed integration into these chromosomes: chromosome 7 in F1, chromosome 12 in F2, chromosome 7 and 10 in F3 (Additional file 1 Table S1). In addition to FACS sorting, we analysed GFP-expression by fluorescence microscopy in blood stages of the 15 subpopulations (a-o) that were obtained from P1 (Figure 2A). Four of the subpopulations contained low numbers of GFP-positive (results not shown), three of which showed low fluorescence intensity (P1f, P1g, P1m) and one, P1e, exhibited a stronger GFP-fluorescence (Figure 4B).

A total of 3 inserts were identified by TAIL-PCR survey in GFP-expressing parasites selected by FACS sorting. Two of these inserts occurred in a direction compatible with GFP expression (Figure 4D and See Additional file 1 Table S1). In addition, 2 inserts were identified from the P1e subpopulation derived by cloning of parent population P1 (See Table Additional file 1 Table S1 for inserts). The identification in a parasite population of one insert that is not compatible with GFP expression may indicate that this population contains parasites with *gfp *inserted into two different loci. FIGE-analysis of this population indeed shows evidence for integration into two loci, one in chromosome 6 and the other in one of the chromosomes of group 9-11 (Figure 4C). We determined transcription of *gfp *by Northern analysis in blood stages of the three FACS-sorted and P1e populations and in all parasites gfp transcripts were detected (Figure 4E). Transcription of gfp insert into the 5'UTR of gene [GeneDB [34]:PBANKA_062360] (population F1/F3) was confirmed by RT-PCR (Figure 4F) demonstrating that our *piggyBac *approach had successfully trapped the promoter of this gene which belongs to the *bir *multi gene family. Interestingly, PCR analysis showed that the size of the RT-PCR amplified *gfp*-transcript in F1/F3 was smaller in comparison with the PCR-amplified genomic fragment (Figure 4F). Sequencing of the RT-PCR amplified product revealed that the smaller size was due to the unexpected removal of an intron in the *piggyBac *5'ITR, not affecting the CDS of *gfp *(Additional file 4 Figure S2). This splicing event shows that the endogenous *piggyBac *5'ITR can be recognized by the splicing machinery of *P. berghei*.

## Discussion

We have evaluated the efficiency and insertion characteristics of the *piggyBac *transposable element in the malaria parasite *P. berghei*. Insertional mutagenesis approaches have been widely used for genome characterization and transposon-mediated mutagenesis has become a powerful molecular genetic tool for eukaryotic transgenesis [46-50]. It has recently been shown that genomic insertional mutagenesis using *piggyBac *combined with a phenotypic screen for attenuated growth of the blood stages provided an effective tool for functional analysis of *Plasmodium *genes [19]. Assuming that appropriate phenotypic screens can be devised, the availability of additional forward genetic technologies holds the great promise for large scale analysis of the function of the many 'hypothetical' *Plasmodium *proteins. Therefore, in this study we adapted the *piggyBac *system used for *P. falciparum *to *P. berghei *and developed it further. *P. berghei*, a rodent malaria parasite, is a frequently used model for the functional analysis of *Plasmodium *genes [10,35,51] and it allows for the analysis of *Plasmodium *gene function both *in vitro *and *in vivo *throughout the complete life cycle.

To obtain *piggyBac *insertions in the *P. falciparum *genome one million parasites were added to erythrocytes preloaded with the transposon donor and the transposase helper plasmid followed by selection of drug resistant parasite populations [18]. In these 'parent populations' the number of insertions was low ranging between 1-14 as identified by inverse PCR or vectorette PCR reactions. Using these methods 177 unique *piggyBac *insertions have been identified in 81 independent transfections [19]. To obtain *piggyBac *insertions in the *P. berghei *genome we used in this study the standard method of transfection of purified schizonts that result in high transfection efficiency of 10^-2^-10^-3 ^when parasites are transfected with plasmids [21,23]. PCR-based detection methods, such as TAIL PCR, have been shown to be highly efficient [52] and the method of choice in other organisms to identify *piggyBac *insertions [53-55]. We used an adapted TAIL PCR method here as traditional PCR was inadequate for these purposes in our hands. Using this TAIL PCR method we were able to detect 35 and 40 inserts, respectively, in the two parent populations (P5, P2) that were obtained by drug selection of 1-5 × 10^6 ^transfected parasites. These calculations indicate that in our studies 16 to 18 times more inserts could be identified per transfection experiment in *P. berghei *than currently reported for *P. falciparum *[19]. It can be expected that a percentage of inserts generated by this approach will not have been identified since the Semi Arbitrary Degenerate (SAD) primers will exercise some specificity and the *P. berghei *genome is extremely AT-rich (< 80%). Furthermore inserts could have been missed since the ability to detect inserts with PCR based methods is highly dependent on the copy number of the insert [56]. The development and use of additional SAD primer sets for Tail PCR in combination with sequencing strategies of increased efficiency/sensitivity might therefore lead to an increased number of identifiable insertions in the parent populations. In the subpopulations and cloned lines that were obtained from the parent populations by infection of mice with 1-5 parasites we estimated that we were able to identify approximately 65% of the inserts by Tail PCR. This is based on the comparison of the number of visible inserts detected in FIGE-separated chromosomes and the inserts identified by TAIL PCR. In total we have identified 127 inserts by TAIL PCR in parasites of only 2 transfection experiments (P2, P5), indicating that *piggyBac *integrates efficiently into the genome of *P. berghei *and that this model permits the generation of *piggyBac *insertion events significantly more efficiently than the human parasite, *P. falciparum*.

Analysis of the insert sites in the *P. berghei *genome showed that insertion occurred exclusively in the expected TTAA insertion site. Like in *P. falciparum *we found neither a (strong) bias for insertion into a particular chromosome nor a preference for insertion into transcribed/expressed genes indicating a random distribution of inserts. This is in contrast to *piggyBac *insertion into the genome of several other organisms, including mouse, zebrafish, *Schistosoma*, *Drosophila *and mammalian cell lines, where insertions predominantly occur into actively transcribed genes [57-60]. Interestingly we observed a slight insertional bias with regard to the sequence directly flanking the TTAA sequence with a slight preference of T's up- and A's downstream of the insertion site, respectively, which has also been observed in *P. falciparum *[19]. On the other hand, we found no preference for insertion within or outside CDS, whereas in *P. falciparum *an increased number of insertions have been observed in the 5'UTR regions, which might also reflect preferential insertion into transcriptionally active genes or subtle differences in genome organization. The fact that *piggyBac *insertion into the *P. berghei *genome is for the most part a random process is important for the further development and application of this technology for larger scale forward genetic approaches.

As with *P. falciparum *we found that in the absence of transposase the *piggyBac *inserts remained stably integrated at the insertion sites even during prolonged periods of asexual multiplication (84 mitotic divisions in a 3 week period). It has been shown that *piggyBac *inserts can remobilize in genomes when transposase is present and several studies have estimated the rate of *transposon *remobilization [43,61,45,60-64]. When we introduced transposase stably into the genome under control of the *ama-1 *promoter remobilization of *piggyBac *inserts was detectable during blood stage asexual multiplication of cloned parasite lines. The observed rate of remobilization seems to be low as the majority of parasites before and after the period of asexual multiplication showed the same insert as judged by analysis of FIGE-separated chromosomes. In the three clones of parasite population 5.i we detected a total of 7-10 unique inserts in parasites that had multiplied for a period of 3 weeks (from the start of the cloning procedure). If we assume as above that we detected 65% of the inserts by TAIL PCR the rate of remobilization in these populations is around 15% per mitotic division (7-10 inserts per 84 mitotic divisions).

Remobilization might actually offer benefits by increasing the number of unique inserts in an experimental population towards the desired saturation levels of mutagenesis [60]. *For P. falciparum *it has been calculated that ~15.000 mutations/inserts will represent about 50% saturation and obtaining such a level of saturation is seen as a difficult but realistic possibility for the *P. falciparum *genome [13]. Remobilization could help to significantly reduce the number of transfections necessary to produce true saturation mutagenesis in a population. For instance, while a 50% coverage library would require ~380 individual transfections using the transient transposase expression strategy (~50 detectable transpositions per transfection) the same level could be obtained with far fewer transfections in parasites containing a stably expressed transposase. Since we observed a 7-10× increase in inserts during 3 mechanical blood passages as a result of remobilisation, a comparable 50% coverage could be obtained by as few as ~50 transfections that are passaged for a period of 3 weeks in mice. The integrated transposase in our experiments is controlled by the *P. berghei ama-1 *promoter, which is active only briefly in the schizont stage. Remobilisation will be especially beneficial if remobilisation can be controlled by regulating transposase activity. Encouragingly the use of inducible expression systems has been shown to greatly improve control of *piggyBac *insertion and remobilization rates in other organisms [24].

*PiggyBac *insertion into CDS or 5'UTRs of genes may provide indirect evidence that the gene is essential for blood stage development. Therefore the data on the location of inserts from large scale *piggyBac *mutagenesis experiments can provide additional evidence for the dispensability of *Plasmodium *genes for blood stage development, information that will be of use for example for validation of drug and vaccine targets. We confirmed the non-essential nature of two of the genes interrupted by *piggyBac *by standard targeted gene deletion. Of these *pb41 *is an orthologue of *pf41 *that encodes a GPI anchored protein found on the merozoite surface and as such as been proposed as a vaccine candidate. Strategies of vaccination targeting non-essential proteins have been attempted in the human infectious parasite *P. knowlesi *in the past and resulted in variant parasites that escaped the vaccination regime. In some cases the escaped parasites failed to express the target antigen [65]. Therefore, knowledge of the essential nature of a protein proposed as a vaccine candidate is potentially significant. The use of the model *P. berghei *to determine the essential nature of conserved genes is relevant due to the relative ease of genetic manipulation in this system and in the cited example we have subsequently learned that PF41 is non-essential in *P. falciparum *(B. Crabb, personal communication).

We have therefore deposited all genes with *piggyBac *inserts in the CDS or in the 5'UTR region (500 bp from the start ATG) in the publically accessible database of genetically modified mutant parasites, RMgmDB [42] and this information on *piggyBac *insertion is linked to the information on individual genes in PlasmoDB [36] and GeneDB [34]. In addition, we demonstrate that *piggyBac *insertion can be used to identify promoters that are active during blood stage development. FACS sorting of GFP expressing blood stage parasites appears to be an efficient method to collect parasites that have GFP inserted downstream of an active gene promoter region (i.e. 5'UTR) initiating GFP expression. One such identified insert was located in the promoter region of a member of the *bir *multigene family [GeneDB [34]:PBANKA_062360]. FACS analysis of blood stage GFP expression of this FACS-sorted population, demonstrated a pattern of GFP expression that is highly comparable to expression of BIR proteins tagged with either GFP or mCherry, specifically showing highest levels of expression in maturing trophozoites and schizonts (results not shown). The most significant application of random mutagenesis is the ability to perform forward genetic screens to select mutants of a desired phenotype. Recently the feasibility of such an approach has been shown for *P. falciparum *by screening for mutants with attenuated growth of the blood stages. This relied upon parasite cloning and phenotype characterisation soon after *piggyBac *integration and screening method resulted in the identification of several parasite genes and pathways critical for intra-erythrocytic development [19]. Such an approach is applicable to both *P. falciparum *and *P. berghei *offering the possibility to develop and apply forward genetic screens for additional and important phenotypes such as virulence, drug resistance, commitment to and successful completion of sexual development. In addition, phenotypic screens might be developed during mosquito transmission and pre-erythrocytic development. However, bottlenecks in parasite numbers during transmission in and out of the mosquito may reduce the efficiency of selecting the desired mutants from a large pool of *piggyBac*-mutants. For *P. berghei *efficient methods exist for production of gametocytes and ookinetes and production of mosquitoes containing large numbers of oocysts (> 500) and salivary gland sporozoites can relatively easily be scaled up. However, application of efficient phenotype screens during liver stage development will require the development of more efficient *in vitro *cultivation systems for the analysis of sporozoites into viable blood stage merozoites.

## Conclusions

This study shows that *piggyBac *is an efficient and random transposon in *P. berghei*. In addition, *piggyBac *is able to remobilize from genomic *P. berghei *insertion sites, which could, with further development facilitate the generation of saturated mutagenesis lines for use in forward genetic screens.

## Methods

### Generation of the *piggyBac *donor and the helper plasmids for transient expression of transposase

The pXL-BACII-GFP-HDHFR *piggyBac *transposon donor plasmid (pL1302) was created by cloning a *gfp*-expression cassette without promoter region and a *hdhfr *selectable marker cassette into the minimal *piggyBac *vector pXL-BACII [28]. The *gfp*-expression cassette, containing *gfpm3 *and the *3'pbdhfr/ts *was excised from plasmid pL0024 by *BamHI/EcoRI *digestion while digesting the pXL-BACII vector with *BglII/EcoRI *resulting in the intermediate plasmid pXL-BACII- *gfpm3-3'pbdhfr *flanked by *XhoI/EcoRI *restriction sites. The *hdfr *selectable marker cassette (*5' pbeef1aa-hdhfr-3'pbdhfr*) was excised from plasmid pL0009 with *EcoRI *digestion and subcloned into the intermediate pXL-BACII- *gfpm3-3'pbdhfr *plasmid, generating the final piggyBac donor plasmid, pXL-BACII-*gfp-hdhfr*. (pL1302). The pHTH transient helper plasmid (pL1301) was constructed by first excising the *piggyBac *transposase coding sequence from the pHTH plasmid used for *P. falciparum *[18]; kindly provided by Bharath Balu) with *BamHI *and cloned into the intermediate plasmid pL0011 under the control of *5'-pbeef1aa *and *3'pbdhfr*, resulting in plasmid pL1303. The *5' pbeef1aa-transposase-3'pbdhfr *cassette was excised from pL1303 with *BamHI *digestion and subcloned into the plasmid pL0004, generating pHTH_pb _(pL1301) for transient transposase expression. The plasmids pL0004, pL0009, pL0011 and pL0024 can be obtained from MR4 [66].

### Construction of a plasmid to generate a transgenic *P. berghei *line stably expressing transposase

To stably integrate the *piggyBac *transposase gene into the *P. berghei *genome plasmid pL1307 was generated. The transposase coding sequence was excised from the pHTH plasmid (see above) with *BamHI *and cloned into the expression plasmid pL0010 (MR4; [66]), which contains the *toxoplasma gondii dhfr-ts *encoding selectable marker cassette and the *dssu-rrna *target sequence for single cross-over integration into the non-essential *c/d-rrna *locus locus. The transposase was cloned with *BamHI *into the *5'pbama1 - 3'pbdhfr *expression cassette of pL0010.

### Transfection and selection of parasites

Transfection, selection and cloning of mutant parasite lines were performed as described [21]. For experiments for transient transfection of parasites with the transposase-containing helper plasmid, circular donor plasmid pL1302 and helper plasmid pL1301 were mixed in a 1:1 (exp. 1056) or 1:2 (exp. 1056) ratios using 5 μg donor plasmid per transfection prior to transfection of the parasites (*P. berghei *ANKA, cl15cy1). Selection of resistant parasite populations after transfection was performed by treatment of the infected mice with pyrimethamine. As a control parasites were transfected with 5 μg donor plasmid (exp 1057). For generation of the transgenic line containing transposase stably integrated into the genome, parasites (*P. berghei *ANKA, cl15cy1) were transfected with plasmid pL1307 after linearization with *Apa-1*. Selection and cloning of transgenic parasites were performed as described [21]. One clone, 1042cl1 (TPS_ama1_) was used for further experiments. After transfection of TPS_ama1 _with donor plasmid pL1302 a resistant parasite population (1182) was selected by treatment with WR9221 [67]. The genotype of transfected parasites was analysed by standard PCR analysis and Southern blot analysis of digested genomic DNA or of FIGE separated chromosomes [23].

### Analysis of transcription: Northern blot and RT-PCR analysis

RNA extraction and Northern blot analysis was performed according to standard methods [68,69]. RNA of mixed blood stages was hybridized to a *gfp*-probe that was PCR amplified from plasmid pL1302 using the primer set 3552-3553 (Additional file 5 Table S3) that is specific for the *gfp *coding sequence (CDS). As a loading control, the ethidium bromide stained gel was used. RNA used for RT-PCR analysis was first digested with *DNaseI *for 1 hour, followed by phenol/chloroform extraction. Reverse transcription was performed using SuperScriptIII™ (Invitrogen) according to the manufacturer's instructions after splitting the RNA into two samples, one to which reverse transcriptase was added (+RT) and one without (-RT). RT-PCR was performed using primer 4571 targeting a *P. berghei *genomic sequence located 117 bp upstream of the confirmed *piggyBac *insertion site in 5' UTR of the *bir *gene [GeneDB [34]:PBANKA_062360]. Primer 4571 was used in combination with primer 3209, located in the *gfp *CDS of the *piggyBac *insert (442 bp from the terminal end of the 5'ITR; see Additional file 5 Table S3 for primer sequences). The expected PCR product size on gDNA using the primer pair 4571/3209 is 559 bp. As a control for reverse transcription and *DNaseI *digestion, the gene [GeneDB [34]:PBANKA_133840] was amplified from both cDNA and genomic DNA with the primer set 3902/3903 located on either side of two confirmed introns (expected sizes are 530 bp on gDNA and 232 bp on cDNA). Thirty-five PCR amplification cycles were performed with Amplitaq DNA polymerase (Roche) using 4 mM MgCl_2 _and an annealing temperature of 50°C and 1 minute extension time at 72°C.

### FACS sorting and analysis of GFP expressing parasites

For FACS sorting of GFP-positive parasites, we collected 10 μl of infected tail blood (with a parasitemia of 3%) in complete culture medium from mice infected with parasites from exp. 1056 (see above). GFP-positive cells were sorted on the FACSAria Cell Sorter (Becton Dickinson). Selection of blood cells on forward/side light scatter and filter settings for detection of GFP-fluorescence was performed as described [70]. GFP-positive cells were sorted using three different gates as shown in the Results Section (Figure 4A) and sorted cells (50-300 cells per sorting experiment) were collected in ~300 μl complete culture medium at room temperature. This cell-suspension was injected intravenously (tail vein) into a single mouse. 7-8 days after injection, parasites were collected from the mice and were analysed for GFP expression. GFP-fluorescence intensity of live blood stages was examined after staining with Hoechst-33258 [71] using a Leica-fluorescence MDR microscope and pictures recorded using a DC500 digital camera.

### Thermal Asymmetric Interlaced (TAIL) PCR and identification of insertion sites

To identify *piggyBac *inserts, two sets of each three primers specific for the *piggyBac *5' and 3'ITR regions (Additional file 5 Table S3) were used successively in combination with 8 Semi-Arbitrary Degenerate (SAD) primers (Additional file 5 Table S3) with 140 to 192 fold degeneracy. Each SAD primer contains a *Sau3Ai *site (4 with GATC and 4 with the reverse complement CTAG at the 3' end) since this sequence is widely distributed in the *P. berghei *genome (approximately 2.3 × 10^4 ^occurrences identified by text searches against all contigs present in PlasmoDB [36]. The SAD primers were designed to reflect the variability surrounding the GATC sites in *P. berghei *from a multiple alignment of around 1100 genomic *P. berghei *sequences obtained from PlasmoDB [36]. TAIL-PCR was performed as described [52,72] with some modifications of the protocol (See Additional file 6 Table S4 for the different conditions used for TAIL-PCR). Briefly, eight primary TAIL-PCR reactions were performed, each with one of the SAD primers (3649-3656) and in combination with either the *piggyBac *5'ITR primer 3202 or the 3'ITR primer 3726. After the initial PCR reaction, products were diluted 1:2000 with distilled water and used in the secondary reaction, containing the *piggyBac *primers 3203 (5'ITR) or 3727 (3'ITR) together with the same SAD primer as used in the primary reaction. The secondary PCR products were diluted 1:500 and used in the tertiary reaction mixture. The tertiary PCR reaction was performed with *piggyBac *primers 3204 (5'ITR) or 3728 (3'ITR) again with the same SAD primers as before. The tertiary amplification fragments were visualized through electrophoresis in 1% agarose gels, where the average fragment size was usually between 300 and 500 bp. Immediately after the tertiary reaction, the reaction mixture was purified using a PCR-purification kit (Roche Applied Science, Germany) according to manufacturers instructions. Subsequently the reaction mixture (containing several TAIL PCR products) was ligated into the TOPO TA vector (Invitrogen) and transformation and blue/white screening was performed according to manufacturer's instructions. DNA from 15 to 20 of positive colonies were analysed by sequencing. The insertion site in the genome of the *piggyBac *cassette was determined by BLASTN (vs. PlasmoDB [36] and GeneDB [34] databases) analysis of the sequences outside of the *piggyBac *cassette followed by precise identification of the insertion site using BioEdit alignment and mapping (using Artemis) of each TAIL-PCR sequence to the closest gene. If an insertion occurred more than 1000 bp from the CDS of a gene, it was not considered to be located in its (potential) 5' or 3'UTRs. For comparison of characteristics of the *piggyBac *insertion sites, 254 randomly TTAA sites and flanking regions were extracted from *P. berghei *genomic sequences (obtained from the Sanger Centre [34]). Visualization of the TTAA insertion site and flanking site conservation was performed with Weblogo 3.0 [73].

### Expression profiles of genes with piggyBac insertions

Using the Boolean operators, implemented in the PlasmoDB site [36], sets of genes with proteome/EST evidence were generated using the databases implemented on the PlasmoDB site [36] including published *P. berghei *gametocyte and proteomes from all developmental stages [3,7]. All datasets were converted to the updated gene models available (released by Sanger Centre as of 15 April 2010) and a comparison was made between all expressed/transcribed genes and genes with *piggyBac *insertions for each set.

### Targeted disruption of *p41 *and *metacaspase2*

Two replacement DNA constructs were made for targeted disruption of *p41 *[GeneDB [34]:PBANKA_100260] and *metacaspase2 *[GeneDB [34]:PBANKA_130230] by double cross-over homologous recombination. Details of primers used to generate double-cross over replacement constructs (i.e. to introduce 'targeting regions' in the replacement construct pL0001; (See MR4 [66] and also Additional file 3 Figure S1 and Additional file 5 Table S3). The *p41 *gene has been disrupted in the ANKA reference line cl15cy1 [21] and *metacaspase2 *has been disrupted in the GFP-expressing reference line of the ANKA strain (line 507cl1, identifier RMgm-7 in [42]). Transfection, selection and cloning of mutant parasites was performed as described [21]. Correct integration of the constructs was analysed by diagnostic PCR and Southern analysis of FIGE-separated chromosomes and transcription by standard Northern analysis of mRNA collected from mixed blood stage parasites [21]. Primers for amplification of the probes used for Northern analysis are shown (See Additional file 3 Figure S1 and Additional file 5 Table S3). As a loading control Northern blots were hybridized with primer L644R that hybridizes to the blood stage large subunit ribosomal RNA [74].

## Authors' contributions

Conceived and designed the experiments: JF, BF, SMK, CJJ, APW. Performed the experiments: JF, BF, SMK, CJJ, JR, OK. Analysed the data: JF, BF, SMK, OK, CJJ. Contributed reagents/materials/tools: JA. Wrote the paper: JF, CJJ, BF, SMK, JA, APW. All authors read and approved the final manuscript.

## Supplementary Material

Additional file 1 **Description of *piggyBac *insertions**. Column 1: Origin of insertion described as: Parental population.clone.subclone (See main text for description). Column 2: Type of insertion in relation to CDS: 5' UTR, CDS, 3'UTR or more than 1 kb away from the nearest CDS (> 1 kb from CDS). Column 3: Insertion site distance to start ATG of CDS (for 5' UTR and CDS insertions) or from the end (stop codon) of the CDS (for 3' insertions). Column 4: Percentage of locus disrupted (for CDS insertions only) and calculated as the percentage of the locus (including introns) occurring after the insertion site. Column 5: Pb locus identifier as used by the Sanger Center (two first digits indicate chromosomal location on the 14 *P. berghei *chromosomes). Column 6: Insertion site sequence (TTAA) with the adjacent 20 5' and 3' nucleotides. Column 7: Gene description as provided by the Sanger Center. Click here for file

Additional file 2 **A characteristic of *piggyBac *inserts in 5'UTR and coding sequences (CDS and introns) of the *P. berghei *genome**. Column 1: Type of insertion in relation to CDS: 5' UTR or CDS. Column 2: Pb locus identifier as used by the Sanger Center (two first digits indicate chromosomal location on the 14 *P. berghei *chromosomes). Column 3: Identifier for *P. falciparum *orthologs. Column 4: Gene description as provided by the Sanger Center. Column 5: Targeted disruption attempted (See column 8 for reference to study). Column 6: Exclusive expression in gametocytes/oocysts/sporozoites ([7,8]). Column 7: Other details regarding gene. Column 8: References to targeted disruption or expression of gene. Column 9: Origin of insertion described as: Parental population.clone.subclone (See main text for further description). Column 10: RMgmDB (RMgmDB; [42,22]) number assigned to disruption. Click here for file

Additional file 3 **Generation of parasites lacking expression of P41 (Δ*p41*) or Metacaspase2 (Δ*metacaspase2*)**. (A) Schematic representation of the construct used for disruption of *p41 *and *metacaspase2*. Correct integration of the construct results in disruption of the genes as shown (replacement locus) and was analysed by Southern analysis of PFG-separated chromosomes and diagnostic PCR (see B and C). See (Additional file 5 Table S3) for primer details. Black boxes: target regions; grey box: *tgdhfr-ts *selectable marker cassette. (B) PCR analysis of correct disruption of *p41*, Northern analysis of transcription in wild type (*wt*) and mutant (318cl1) and Southern analysis of PFG-separated chromosomes. PCRs were performed with primers that amplify the 5' (INT1 and L313) region of the disrupted locus (5' int) or the intact ORF (INT1, INT2), c: Control PCR amplifying the *p28 *gene. Chromosomes were hybridized with the 3'-*dhfr *probe recognizing the *p41*-construct integrated in chromosome 10 and the endogenous *dhfr-ts *gene on chromosome 7. TM4 is a probe recognizing rRNA and used as a loading control. (C) PCR analysis of correct disruption of the *metacaspase2 *and Southern analysis of PFG-separated chromosomes in wild type (*wt*) and mutant (796cl1) (right-hand panel). PCRs were performed with primers that amplify the 5' (INT1 and 313) region of the disrupted locus (5' int) or the intact ORF (INT1, INT2), c: Control PCR amplifying the *p28 *gene. Chromosomes were hybridized with the 3'-*dhfr *probe recognizing the *metacaspase2 *construct integrated in chromosome 10, the endogenous *dhfr-ts *gene on chromosome 7 and the integrated GFP-construct in chromosome 3 of the parent line 507cl1. Click here for file

Additional file 4 **Location of intron within the 5'ITR of *piggyBac***. Green letters: *gfp *CDS. Black underlined letters: piggyBac 5' ITR. Black underlined bold letters: Intron. Click here for file

Additional file 5 **List of primers used in this study**. Left column: primer number. Middle column: Primer sequence. Right column: Description of primer and orientation (F: Forward, R: Reverse). Click here for file

Additional file 6 **TAIL PCR conditions during Primary, Secondary and Tertiary rounds of PCR (adapted from **[75]**)**. Click here for file
