# Supplementary figures and images for: Development of the piggyBac transposable system for Plasmodium berghei and its application for random mutagenesis in malaria parasites
Source: BMC Genomics. 2011 Mar 20;12:155. doi: 10.1186/1471-2164-12-155 (PMC3073922; doi:10.1186/1471-2164-12-155)

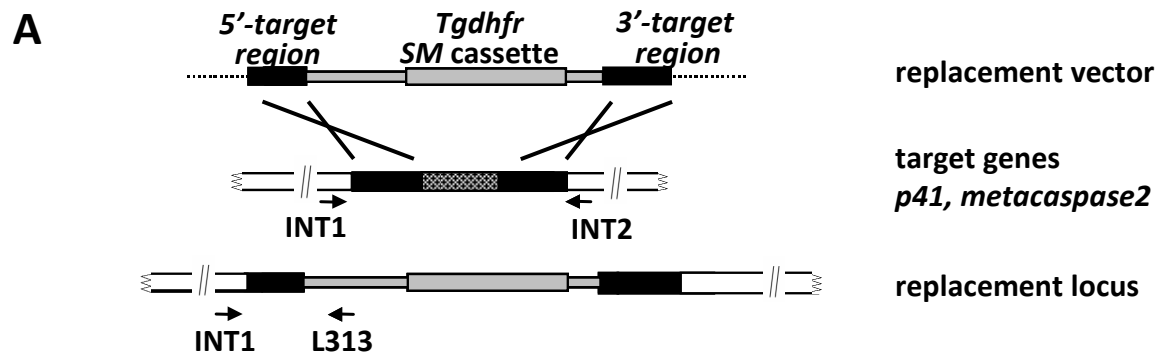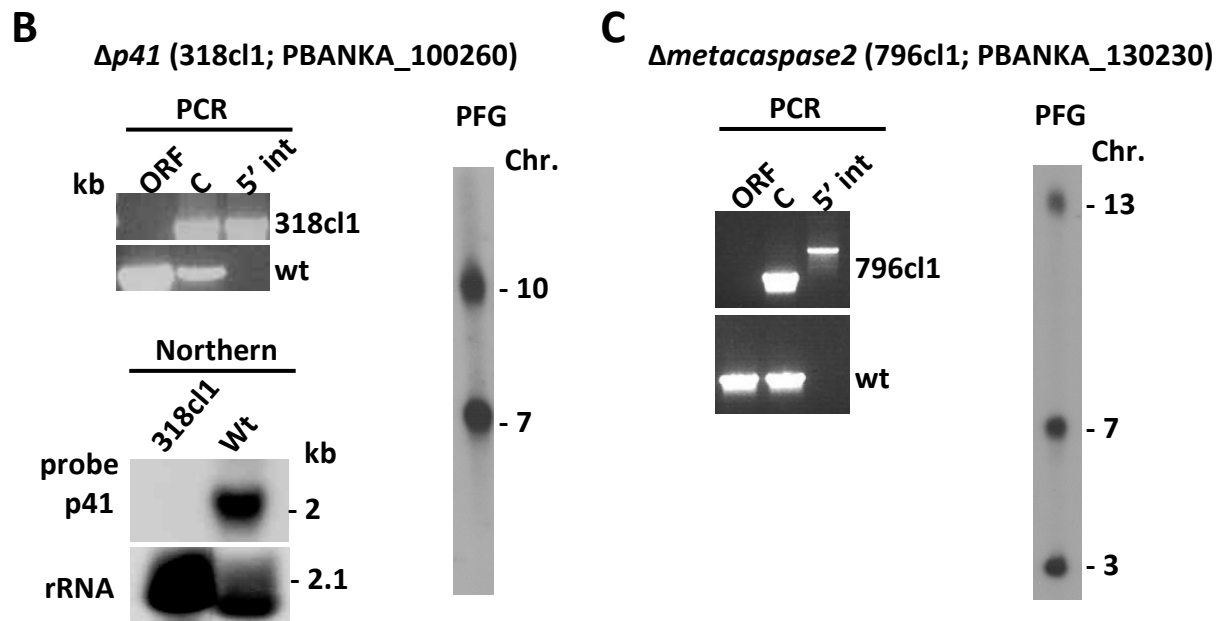

Supplement: Additional file 3 — Generation of parasites lacking expression of P41 (Δp41) or Metacaspase2 (Δmetacaspase2). (A) Schematic representation of the construct used for disruption of p41 and metacaspase2. Correct integration of the construct results in disruption of the genes as shown (replacement locus) and was analysed by Southern analysis of PFG-separated chromosomes and diagnostic PCR (see B and C). See (Additional file 5 Table S3) for primer details. Black boxes: target regions; grey box: tgdhfr-ts selectable marker cassette. (B) PCR analysis of correct disruption of p41, Northern analysis of transcription in wild type (wt) and mutant (318cl1) and Southern analysis of PFG-separated chromosomes. PCRs were performed with primers that amplify the 5' (INT1 and L313) region of the disrupted locus (5' int) or the intact ORF (INT1, INT2), c: Control PCR amplifying the p28 gene. Chromosomes were hybridized with the 3'-dhfr probe recognizing the p41-construct integrated in chromosome 10 and the endogenous dhfr-ts gene on chromosome 7. TM4 is a probe recognizing rRNA and used as a loading control. (C) PCR analysis of correct disruption of the metacaspase2 and Southern analysis of PFG-separated chromosomes in wild type (wt) and mutant (796cl1) (right-hand panel). PCRs were performed with primers that amplify the 5' (INT1 and 313) region of the disrupted locus (5' int) or the intact ORF (INT1, INT2), c: Control PCR amplifying the p28 gene. Chromosomes were hybridized with the 3'-dhfr probe recognizing the metacaspase2 construct integrated in chromosome 10, the endogenous dhfr-ts gene on chromosome 7 and the integrated GFP-construct in chromosome 3 of the parent line 507cl1. [file 1471-2164-12-155-S3.PDF]
