# Supplementary material for: Development of the piggyBac transposable system for Plasmodium berghei and its application for random mutagenesis in malaria parasites
Source: BMC Genomics. 2011 Mar 20;12:155. doi: 10.1186/1471-2164-12-155 (PMC3073922; doi:10.1186/1471-2164-12-155)
Supplement: Additional file 4 — Location of intron within the 5'ITR of piggyBac. Green letters: gfp CDS. Black underlined letters: piggyBac 5' ITR. Black underlined bold letters: Intron. [file 1471-2164-12-155-S4.PDF]

TTATTTGTATAGTTCATCCATGCCATGTGTAATCCCAGCA
GCTGTTACAAACTCAAGAAGGACCATGTGGTCTCTCTTT
TCGTTGGGATCTTTCGAAAGGGCAGATTGTGTGGACAG
GTAATGGTTGTCTGGTAAAAGGACAGGGCCATCGCCAA
TTGGAGTATTTTGTTGATAATGGTCTGCTAGTTGAACGC
TTCCATCTTCAATGTTGTGTCTAATTTTGAAGTTAACTTT
GATTCCATTCTTTTGTTTGTCTGCCATGATGTATACATTG
TGTGAGTTATAGTTGTATTCCAATTTGTGTCCAAGAATG
TTTCCATCTTCTTTAAAATCAATACCTTTTAACTCGATTCT
ATTAACAAGGGTATCACCTTCAAACTTGACTTCAGCACG
TGTCTTGTAGTTCCCGTCATCTTTGAAAAATATAGTTCTT
TCCTGTACATAACCTTCGGGCATGGCACTCTTGAAAAAG
TCATGCTGTTTCATATGATCTGGGTATCTCGCAAAGCAT
TGAACACCATAACCGAAAGTAGTGACAAGTGTTGGCCA
TGGAACAGGTAGTTTTCCAGTAGTGCAAATAAATTTAA
GGGTAAGTTTTCCGTATGTTGCATCACCTTCACCCTCTCC
ACTGACAGAAAATTTGTGCCCATTAACATCACCATCTAA
TTCAACAAGAATTGGGACAACTCCAGTGAAAAGTTCTTC
TCCTTTACTCATGGATCTTAATACGACTCACTATAGGGC
GAATTGGGTACCGGGCCCCCCCTCGAGGTCGACGGTAT
CGATAAGCTTGATAT**CTATAACAAGAAAATATATATAT**
AATAAGTTATCACGTAAGTAGAACAT**GAAATAACAAT**
ATAATTATCGTATGAGTTAAATCTTAAAAGTCACGTAA
AAGATAATCATGCGTCATTTTGACTCACGCGGTCGTTA
TAGTTCAAAATCAGTGACACTTACCGCATTGACAAGCAC
GCCTCACGGGAGCTCCAAGCGGCGACTGAGATGTCCTA
AATGCACAGCGACGGATTCGCGCTATTTAGAAAGAGAG
AGCAATATTTCAAGAATGCATGCGTCAATTTTACGCAGA
CTATCTTTCTAGGG
