# Supplementary material for: Development of the piggyBac transposable system for Plasmodium berghei and its application for random mutagenesis in malaria parasites
Source: BMC Genomics. 2011 Mar 20;12:155. doi: 10.1186/1471-2164-12-155 (PMC3073922; doi:10.1186/1471-2164-12-155)
Supplement: Additional file 5 — List of primers used in this study. Left column: primer number. Middle column: Primer sequence. Right column: Description of primer and orientation (F: Forward, R: Reverse). [file 1471-2164-12-155-S5.PDF]

**Name**	**Sequence 5` to 3`**	**Description and primer orientation**
L313	ACGCATTATATGAGTTCATTTTAC	*p41/mc2* R
L537	TGCTCTAGAATGAATTTTAAATACAGTTTTATT	*p28* (PBANKA_051490) F
L538	TGCTCTAGATTACATTACTATCACGTAAATAAC	*p28* (PBANKA_051490) R
L635	TTTCCCAGTCACGACGTTG	*Transposase* R (5` integration check)
L644R	GAACAAATTACTTCATTCATAGC	3'-*lsu-rrna*
L702	CATGAAGATATCGAGGCGGAG	TM4 antisense ribosomal A oligo
L739	TTTGGATATTTTCATATATG	wt *c-* and *d-ssu-rrna* F
L740	CTAAGGTACGCATATCATGG	wt *c-* and *d-ssu-rrna* R
L741	CGCGGATCCATGCATAAACCGGTGTGTC	*T. gondii dhfr/ts* F
L742	CGCGGATCCGCTAGACAGCCATCTCCAT	*T. gondii dhfr/ts* R
L1249	CC<u>ATCGAT</u>GTATTTGATTTATTAGTTGTGTGTC	*p41* F 5` target region (*ClaI*)
L1250	CCC<u>AAGCTT</u>CATTATGAAAACAGTGTGCTCC	*p41* R 5` target region (*HindIII*)
L1251	G<u>GATATC</u>TAGAACCTTCTGGATGCTTCG	*p41* F 3` target region (*EcoRV*)
L1252	CGC<u>GGATCC</u>AAATAATCACTAGCTTAATAACC	*p41* R 3` target region (*BamHI*) I
L1411	GGCATATGATAGGAGCACACTGTTTTCATAATG	*p41 F (ORF)*
L1412	CGCGGATCCTCAATTAAAAGAGTTTTTAATTAAATCAC	*p41 R (ORF)*
L1478	TCAG<u>GGTACC</u>GAATTATATATGCTTATAAAAGATGTTCACAGC	*metacaspase2(mc2)* F 5` target region (*KpnI*)
L1479	TCAG<u>AAGCTT</u>GAATTATTTAAGTTATTAATATAATTTTCGACG	*metacaspase2(mc2)* R 5` target region (*HindIII*)
L1480	TCAG<u>GAATTC</u>CACTGAGTTATAATGGTTTATTGGAAGGATGCG	*metacaspase2(mc2)* F 3` target region (*EcoRI*)
L1481	TCAG<u>TCTAGA</u>TTATTTCGAATTTGTATATATATGTATGGATTCGC	*metacaspase2(mc2)* R 3` target region (*XbaI*)
L1524	TAACTGTAATAGGTAGACAATG	*p41* F (5` INT1)
L1662	GATTCATAAATAGTTGGACTTG	*Transposase* F (3` integration check)
L1686	GTAAATGATTTAATAATTATAACAAATTTAGTACGC	*metacaspase2(mc2)* F (5` INT1/F ORF)
L1687	GTGAAAATTGAGAGACAGCATAATGAATTAGTACAC	*metacaspase2(mc2)* R (ORF)
3649	TANWTAATAATAWWWWGATC	SAD1
3650	ATAWATTATWWWWAWDGATC	SAD2
3651	ATATAATAHTTAWDWNGATC	SAD3
3652	TATATTAWWAATWNWWGATC	SAD4
3653	TANWTAATAATAWWWWCTAG	SAD5
3654	ATAWATTATWWWWAWDCTAG	SAD6
3655	ATATAATAHTTAWDWNCTAG	SAD7
3656	TATATTAWWAATWNWWCTAG	SAD8
3202	AGATGTCCTAAATGCACAGCGAC	5`ITR
3203	CTCCAAGCGGCGACTGAG	5`ITR
3204	CATTGACAAGCACGCCTCAC	5`ITR
3205	GTCAATGCGGTAAGTGTCACTGA	5`ITR
3206	GTAAGTGTCACTGATTTTGAACTATAACG	5`ITR
3207	GACGCATGATTATCTTTTACGTGAC	5`ITR
3726	CTCGAATTCGATAAAAGTTTTGTTAC	3`ITR
3727	CTCGATATACAGACCGATAAAACAC	3`ITR
3728	CATGATTATCTTTAACGTACGTCAC	3`ITR
3209	CATCTAATTCAACAAGAATTGGGAC	*gfp* promoter trap F
3552	GTGTAATCCCAGCAGCTGTTACAAAC	*gfp* probe F
3553	GTTGAATTAGATGGTGATGTTAATG	*gfp* probe R
3902	GGCACAAAGCAAAGGATCCAACG	PBANKA_133840 cDNA control F
3903	GCTCAATTTTAGTCACAATCTCATC	PBANKA_133840 cDNA control R
4571	ATGCATCAAACCTATATTTAATCAC	PBANKA_062360 R

F: Forward. R:Reverse. Restriction enzyme sites are underlined
