# Supplementary material for: Development of the piggyBac transposable system for Plasmodium berghei and its application for random mutagenesis in malaria parasites
Source: BMC Genomics. 2011 Mar 20;12:155. doi: 10.1186/1471-2164-12-155 (PMC3073922; doi:10.1186/1471-2164-12-155)
Supplement: Additional file 6 — TAIL PCR conditions during Primary, Secondary and Tertiary rounds of PCR (adapted from [75]). [file 1471-2164-12-155-S6.PDF]

Reaction	Cycle number	Thermal condition
**Primary**	1	92°C (3 min), 95°C (2 min)
	10	94°C (30 s), 55 °C (1 min), 72°C (1 min)
	1	94°C (1 min), 25°C (2 min) ramping to 72°C over 2 min, 72 °C (1 min)
	15 [a]	94°C (30 s), 55°C (1 min), 72°C (1 min),
		94°C (30 s), 55°C (1 min), 72°C (1 min),
		94°C (30 s), 35°C (1 min), 72°C (1 min),
	1	72°C (5 min)
**Secondary**	1	94°C (5 min)
	12 [a]	94°C (30 s), 55°C (1 min), 72°C (1 min),
		94°C (30 s), 55°C (1 min), 72°C (1 min),
		94°C (30 s), 35°C (1 min), 72°C (1 min),
	1	72°C (5 min)
**Tertiary**	1	94°C (5 min)
	35	94°C (30 s), 35°C (1 min), 72°C (1 min)
	1	72°C (5 min)

[a] Two nine-segment super cycles, which alternate between two high-stringency and one low stringency cycle.
